# Cinnamonium

**Published:** 1888-04

**Authors:** John Hall


### CINNAMONIUM.

March 7th, 1888.—I may be permitted to introduce my essay by a quotation from Samuel Hahnemann, which, though his precise words are not now at command, was given very much as follows: Whatever a physician may give, either as his own provings of certain remedies or his personal experience, should be of a truthful character; indeed, so impressed was our founder with the importance of this suggestion that he required of all his friends who had proved medicines an oath that they believed, so far as they could decide, that the symptoms so induced in their persons were the legitimate result of the remedy they had been taking and were therefore reliable.

The very same remark applies to ourselves, the feature most recognized among medical men, being a sense of moral responsibility that they are ever treating human beings, about whom there is "nothing hidden which shall not be revealed." It is with this view, as President of this Club, that I urge the utmost care in our cases, that those who may come after us may rely upon our decisions as truth. But as, in truth, questions and cases will come up once in awhile in which we may have neither knowledge nor experience to offer, it becomes us to say but little, though every one must admit that the mere presentation of such cases will do us all good by merely evolving our thoughts on such subjects.

The subject given me is Cinnamonium, about which I have but little personal experience to offer, being by necessity compelled to avail myself of those who have had.

The first remark I have to make is that this remedy, so far as proved, shows a marked tendency on the lymphatic, feeble, and cachectic. With a lax tissue and languid circulation a great tendency to hysteria, and always worse by any lifting or exertion. With these conditions in view a few marked symptoms are given:

1. *Hemorrhage during* or after gestation from lifting, straining, over stretching the arms, or on a false step.

2. Severe hemorrhage in a primipara after the first few pains, when the os has dilated about an inch; the *placenta descending with the head.*

3. Metrorrhagia soon after delivery and not accompanied with plethora.

The character of the flood, whether venous or arterial, not yet known. But the remedy is not sufficiently thought of in 1, *Chronic* METRORRHAGIA ; 2, *Typhoid fever hemorrhage ;* 3, *Hysteria.*

JOHN HALL, SR.

